# Rhythms in longitudinal thalamic recordings are linked to seizure risk

**DOI:** 10.1002/epi.70148

**Published:** 2026-02-16

**Authors:** Xinbing Zhang, Zachary T. Sanger, Thomas Lisko, Steffen Ventz, Robert A. McGovern, Theoden I. Netoff

**Affiliations:** ^1^ Department of Biomedical Engineering University of Minnesota Minneapolis Minnesota USA; ^2^ Department of Neurosurgery University of Minnesota Minneapolis Minnesota USA; ^3^ Division of Biostatistics and Health Data Science University of Minnesota Minneapolis Minnesota USA

**Keywords:** anterior nucleus of thalamus, cycles in epilepsy, deep brain stimulation, seizure forecasting, thalamic recording

## Abstract

**Objective:**

Seizure unpredictability remains a major clinical challenge for people with epilepsy. Previous works have shown that seizure risk is associated with circadian and multi‐day cycles in both brain and physiological signals. However, it remains unclear whether neural activity from deep brain structures such as the anterior nucleus of the thalamus (ANT), the only U.S. Food and Drug Administration (FDA)–approved deep‐brain stimulation (DBS) target for treating medication‐resistant epilepsy, exhibits similar cyclic modulation related to seizures. This study aimed to assess whether long‐term local field potential (LFP) recordings from the ANT exhibit circadian and multi‐day cycles that are associated with seizures that could be used to support seizure forecasting in a retrospective approach.

**Methods:**

Seven participants implanted with the Medtronic Percept PC system for ANT‐DBS underwent continuous at‐home LFP recording of theta/alpha (4–12 Hz) and self‐reported seizure logs. Wavelet and Hilbert transforms were used to identify rhythmic cycles in LFPs. Circular statistics quantified seizure phase‐locking to LFP cycles and patterns estimated from seizure diaries. Gaussian process regression (GPR) models were trained using the instantaneous phase and amplitude of these cycles to forecast short‐term seizure risk.

**Results:**

A total of 7.37 years of at‐home LFP recordings were analyzed. All seven participants exhibited circadian and/or multi‐day cycles in their ANT LFPs, with seizures significantly phase‐locked to some of these cycles. Incorporating the instantaneous cycle amplitude improved seizure forecasting performance across all participants. Moreover, a substantial, although non‐significant, positive correlation between circadian cycle power and seizure frequency was found in most participants, suggesting an elevated seizure risk when circadian cycles are stronger.

**Significance:**

This study demonstrates that long‐term LFP recordings from the ANT reflect rhythmic brain activity linked to seizure risk, and that cycle amplitude provides complementary information about seizure risk than the phase alone.

**Key points**

- Self‐reported seizures were phase‐locked to circadian and multi‐day cycles in local field potential (LFP) power measured in the anterior nucleus of the thalamus.
- Both the instantaneous amplitude and phase of these cycles provided predictive information about seizures.
- Fluctuation of the circadian cycle strength showed positive correlation with seizure frequency in 5 of 7 participants.

## 1 INTRODUCTION

Daily and multi‐day periodicities in seizure occurrence among individuals with epilepsy have been well documented for centuries.^1^, ^2^, ^3^ Seizures are known to cluster at specific times of day and across multi‐day intervals, associated with long‐term factors such as sleep,^4^, ^5^ stress,^4^, ^6^ diet,^7^ and exercise.^8^ These rhythmic patterns provide an opportunity to understand the mechanism underlying seizure occurrence,^9^, ^10^, ^11^, ^12^ and potentially improve seizure forecasting and personalized epilepsy management strategies.^13^, ^14^, ^15^, ^16^, ^17^, ^18^, ^19^

Recent advancements in long‐term monitoring using implantable and wearable devices have revealed that various neural and physiological signals, including interictal epileptiform activity (IEA),^20^, ^21^, ^22^ heart rate,^23^ and electrodermal activity,^24^ are modulated over circadian and multi‐day rhythms. Of note, seizures often cluster at a certain phase of these cycles,^20^, ^23^, ^24^ indicating that tracking multimodal biomarkers could improve seizure forecasting. The first in‐human prospective clinical trial of seizure forecasting using long‐term electroencephalography (EEG) has demonstrated the feasibility of this approach.^25^

The anterior nucleus of the thalamus (ANT) is currently the only U.S. Food and Drug Administration (FDA)–approved deep‐brain stimulation (DBS) target for treating epilepsy.^26^, ^27^ With the advent of the Medtronic Percept DBS system, simultaneous stimulation and recording of local field potential (LFP) at the implant site is now possible.^28^ Using this system, we have recently observed that slow‐gamma oscillations (20–50 Hz) are suppressed by high‐frequency stimulation, indicating that thalamic activity is modulated by the treatment.^29^ A previous clinical study showed that ANT‐DBS modulates circadian and multi‐day cycles of IEA at the seizure‐onset zone (SOZ).^30^ Given that seizures often cluster at certain phases of underlying biological rhythms,^23^, ^24^ monitoring thalamic neural activity may inform when a patient is at a high or low seizure risk. Fluctuations in the amplitude and phase of these cycles may indicate deviations from baseline seizure susceptibility. Therefore, understanding the dynamics of thalamic cycles could offer a potential biomarker for forecasting seizures and personalizing DBS therapy.

In this study, we tested the hypothesis that theta/alpha (4–12 Hz) oscillations in ANT exhibit circadian and multi‐day cycles, and that self‐reported seizures occur at specific phases of these cycles and correlate with the strength of these cycles. All participants were implanted and chronically monitored using the Medtronic Percept system.

## 2 MATERIALS AND METHODS

### 2.1 Subjects

Participants' (*N* = 7) data analyzed in this study have been collected as part of an ongoing clinical trial (ClinicalTrials.gov ID: NCT05493722) approved by the University of Minnesota Institutional Review Board. All participants received the Medtronic Percept PC implantable pulse generator (IPG), which enables simultaneous stimulation and LFP recording. Participants were implanted with either the older Medtronic Legacy leads (Model 3389) or the latest Medtronic Sensight DBS leads (Model B33005) in bilateral ANTs. Participants were included if they had at‐home recordings of theta and alpha (4–12 Hz) Berger band lasting longer than 2 months and provided diaries with time‐stamped seizure events.

### 2.2 At‐home monitoring

The IPG estimates a fast Fourier transform (FFT) of each segment of LFP sampled at 250 Hz, and the 10‐min average power of a predefined 5 Hz frequency band was saved to the memory buffer.^28^ Thus the IPG records 144 LFP samples per hemisphere per day. The Medtronic Percept PC has a 60‐day memory with a first‐in‐first‐out buffer. Recordings were exported in JSON and downloaded at each in‐clinic and at‐home visit, scheduled about every 60 days.

### 2.3 Threshold rejection and discontinuity in recordings

Recorded ANT LFP power was mostly around 1e3 μV^2^/Hz. However, occasional overvoltage samples exceeded 1e7 μV^2^/Hz and showed no correspondence with participant‐reported events. As their relevance could not be verified, samples above 10^6 μV were rejected. Recordings were converted to power (dB), and a second‐order autoregressive moving‐average model was used to interpolate missing data. The lead contact impedances were linearly interpolated for each 30‐day period.

### 2.4 Seizure diary

Seizure events were self‐reported by participants using the Medtronic handheld and/or a written seizure diary. Some participants reported a single event multiple times due to unfamiliarity with the handheld. To avoid repetitive event reports, seizures with an inter‐seizure interval of less than 10 min were excluded. In addition, we were only interested in forecasting the leading seizures of potential clusters. To identify seizure clusters, the distribution of log‐transformed inter‐seizure intervals was modeled using a Gaussian mixture model (GMM). A bimodal distribution is expected when seizure clusters are present, as clustered events tend to have much shorter inter‐seizure intervals than others, forming a distinct peak in the histogram. A distribution was classified as bimodal if a two‐component GMM had a lower Bayesian information criterion, indicating a better fit to the data, than a single‐component model. The component corresponding to shorter intervals was excluded from the analysis. In addition, seizures reported during an LFP recording gap were also excluded from the analysis. To align seizure occurrence with the 10‐min LFP recordings, each seizure time was adjusted to the nearest subsequent LFP sample time.

### 2.5 Cycle identification and extraction

To identify cycles in LFP recordings, a scalogram estimated using a Morlet wavelet transform was averaged across time to obtain a power spectrum. To determine the significance of cycles, 1000 normally distributed surrogate LFPs were generated. A local peak was considered statistically significant if its power exceeded the 95th percentile of the surrogate distribution. Circadian and multi‐day cycles were rounded to the nearest integer days. For example, a cycle peak at a 5.1‐day cycle would be rounded to a 5‐day cycle. Cycles with a period longer than half of the longest continuous recording segment, without any gaps longer than 24 h, were excluded from the analysis.

After identifying significant cycles, a bandpass filter was used to extract the signal at each cycle frequency with a ±33% window width. For example, to extract the circadian cycle (1 cycle/day), the signal was bandpass‐filtered between 0.75 and 1.33 cycles/day. All filters used an infinite impulse response (IIR) to ensure time‐causal filtering. Data were processed using MATLAB (R2024a; The MathWorks, Inc., Natick, MA). The *bandpass()* and *filter()* functions were used to design and apply an IIR filter. The *hilbert()* function was used to compute the analytic signal, from which instantaneous cycle phase and amplitude were extracted.

In addition, circadian and multi‐day patterns in the seizure diary were estimated by performing the same analysis but using simulated sinusoids with periods from 1 day to one‐fifth of the recording length.^13^

### 2.6 Seizure phase‐locking analysis

To examine seizure timing relative to LFP cycles, we extracted the instantaneous cycle phase at the time of reported seizure events. To quantify the strength of phase‐locking of seizures to LFP cycles, we computed the re‐normalized mean resultant vector (*R*‐value) described in Andrzejak et al.^31^ Statistical significance was assessed using the Rayleigh test via the *circ_rtest()*.^32^ Only LFP cycles or seizure diary–derived patterns that showed significant phase‐locking were included in subsequent seizure forecasting analyses.

### 2.7 Seizure risk modeling and forecasting

Gaussian process regression (GPR) is a non‐parametric Bayesian approach that can be used to model the distribution over functions mapping input (LFP cycles) to seizure probability.^33^ Unlike traditional regression methods, GPR learns flexible patterns directly from each participant's LFP cycles and seizure events. The LFP cycles' instantaneous phase and amplitude, estimated using the Hilbert transform at each 10‐min timestamp, were used to model seizure risk. We evaluated three GPR‐based models that were trained to capture time‐varying seizure probability as a function of identified cycles:

- Model 1 used only the LFP cycle phase.
- Model 2 incorporated both the LFP cycle amplitude (in dB) and phase.
- Model 3 used the phase of simulated sinusoids based on cycles found in the seizure diary without neural recordings, as described in Karoly et al.^13^

Seizure risk was forecasted every 10 min. Due to the limited number of seizure events, each model included no more than two cycles for seizure risk modeling and forecasting. If both hemispheres exhibited circadian LFP cycles phase‐locked to seizures, the cycle with the higher *R*‐value to seizures was used. In addition, the multi‐day cycle with the highest *R*‐value was included. Similarly, Model 3 uses the diary‐based circadian cycle and the multi‐day cycle with the highest *R*‐value to model seizure risk.

For the GPR model, a Matern 3/2 kernel with automatic relevant determination was used. To ensure numeric stability during model optimization, the length scale parameters were initialized to 2 and 0.5 for modeling cycle amplitude and cycle phase, respectively. Model performance was evaluated using five‐fold cross‐validation with folds split chronologically to preserve the temporal structure of the recordings. Each fold uses 20% of the data for testing, regardless of the number of seizures, without random shuffling or stratification by seizure samples. To reduce the effects of an imbalanced dataset in model training, we randomly sampled non‐seizure timepoints to maintain a 1:3 seizure to non‐seizure sample ratio. Cycle amplitude and phase inputs were *z*‐scored within each fold.

Forecasting performance was quantified using the area under the curve (AUC) of the sensitivity versus proportion of corrected time in the warning curve.^22^ To determine better than chance forecasting, 200 surrogates with randomly shuffled seizure‐to‐seizure intervals were used for training, and the 95th percentile was used as the threshold.

### 2.8 Circadian cycle modulation and seizure frequency

To examine the longitudinal change in the circadian cycles, we estimated the monthly power of circadian cycles in each hemisphere. This was done by averaging the power of the Morlet wavelet transform centered around the circadian frequency (±33% bandwidth). To account for impedance‐related variations in signal amplitude, circadian cycle power was normalized by the average impedance of the two recording contacts per hemisphere (Sensight: Left‐0 & 7, Right‐8 & 15; Legacy: Left‐0 & 3, Right‐4 & 7).

Monthly seizure frequency was calculated from self‐reported seizure logs prior to the seizure cluster removal described in Section 2.4. Association between seizure frequency and circadian power in the dominant hemisphere (i.e., the hemisphere with stronger circadian modulation) was assessed using two approaches: (1) a linear regression model fitted across all participants, with data z‐scored within participant; and (2) Pearson correlation coefficients computed within each participant's data. To reduce noise, a 3‐point moving average was applied to the circadian cycle power and monthly seizure frequency in the within‐participant analyses.

### 2.9 Mutual information permutation test for circadian modulation

To evaluate whether LFP recordings exhibit meaningful circadian modulation beyond random fluctuations, we computed the mutual information between time of day and hourly averaged LFPs. Significance was assessed using the 95th percentile of a distribution generated from 1000 time‐shuffled permutations. This analysis served as a complementary validation to the method described in Section 2.5.

## 3 RESULTS

### 3.1 ANT LFP recordings

Seven participants, four (P1–P4) implanted with the Medtronic Sensight leads and three (P5–P7) implanted with the Legacy leads, were included in the study. A total of 7.37 years of at‐home LFP recordings were analyzed. Demographic information of the population is shown in Table 1. Possible seizure clusters were found in four participants' seizure diaries (Figure S1). Only the leading seizure of each cluster was included in the seizure forecasting analysis.

**TABLE 1:** Participant demographics.

Participant ID	Sex/age	Recording length in days (% missing data)	Reported seizure # (after cluster removal)	Seizure frequency /month (after cluster removal)	Seizure types	Relevant history/imaging findings/prior procedures	Lead type	Other devices
P1	M 30s	378 (0.4)	28 (17)	2.2 (1.3)	Focal impaired consciousness Focal impaired consciousness→tonic–clonic	Traumatic brain injury; primary left frontal encephalomalacia, smaller areas of right temporal and occipital encephalomalacia	Sensight	No
P2	M 50s	535 (6.2)	123 (123)	6.9 (6.9)	Focal preserved consciousness Focal impaired consciousness with observable manifestations: distal and proximal automatisms Generalized tonic–clonic	Left inferior temporal gyrus topectomy; left inferior parietal/occipital topectomy	Sensight	VNS (OFF)
P3	F 20s	598 (8.8)	26 (26)	1.3 (1.3)	Focal preserved consciousness Focal impaired consciousness	Perinatal ICH; bilateral parietoccipital encephalomalacia	Sensight	No
P4	M 50s	545 (0.5)	60 (44)	3.3 (2.4)	Focal impaired consciousness Focal impaired consciousness with observable manifestations: hyperkinetic behavior Focal impaired consciousness→generalized tonic–clonic	None	Sensight	VNS (ON)
P5	F 40s	483 (1.6)	415 (319)	25.8 (19.8)	Focal preserved consciousness with observable manifestations: aphasia Focal impaired consciousness with observable manifestations: automatisms	Right temporal lobectomy	Legacy	PaceMaker (ON)
P6	F 30s	130 (0.2)	64 (54)	14.8 (12.5)	Focal preserved consciousness Focal impaired consciousness Focal impaired consciousness→generalized tonic–clonic	Parietooccipital encephalomalacia	Legacy	RNS (ON)
P7	F 40s	156 (23.0)	21 (21)	4 (4)	Focal preserved consciousness Focal impaired consciousness Focal impaired consciousness→generalized tonic–clonic	None	Legacy	VNS (ON)

Abbreviations: F, Female; M, Male; RNS, responsive neurostimulation; VNS, vagus nerve stimulator.

**FIGURE 1:**
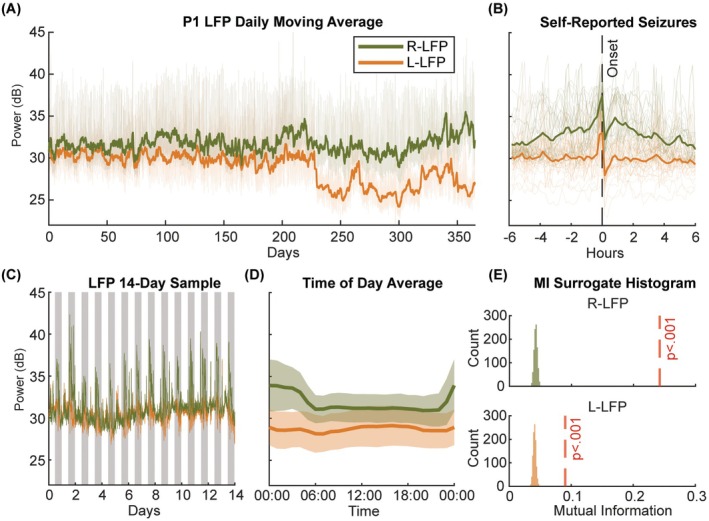
Participant 1: example of LFP (local field potential) power recording. P1's 10‐min average of the theta/alpha band LFP power recordings. (A) Daily moving averages of LFP powers in each hemisphere (green: Right ANT (anterior nucleus of thalamus); orange: Left ANT). (B) Twelve‐hour LFP recordings aligned to self‐reported seizures. The *x*‐axis shows time relative to seizure onset. (C) Recording for the first 14 days. Gray shading marks time from 8 p.m. to 8 a.m. each day. (D) LFP power averaged by time of day, highlighting consistent circadian modulation in the right ANT. (E) Histograms from the mutual information permutation test assessing circadian modulation. The red dashed line marks the mutual information using the actual data and the histogram shows the distribution of 1000 permutation surrogates. Both hemispheres of P1 exhibited significant circadian modulation.

Power fluctuations and rhythmic patterns of ANT LFP were observed in all participants. In P1, a characteristic pattern of increased LFP was found around self‐reported events (Figure 1B). Similar patterns were observed in some other participants (Figure S2), suggesting that ANT LFP recordings may capture seizure‐relevant neural activity. A 14‐day sample revealed the circadian rhythm in P1 (Figure 1C), whose right ANT LFP power increased during the night and decreased during the day. A similar, but weaker, pattern was seen in the left hemisphere (Figure 1D). The mutual information between time and LFP power indicated significant circadian modulation in P1's two hemispheres (*p* < .05, permutation test, Figure 1E). Similarly, six out seven participants, all except P2, showed significant circadian modulation of LFP powers in at least one hemisphere (*p* < .05; Table S1). Please note that the abrupt change in P1's L‐LFP around 220 days was likely due to the change of the DBS stimulation setting (Figure 1A), which was explored previously in a case study.^34^

### 3.2 Circadian and multi‐day cycles in ANT

**FIGURE 2:**
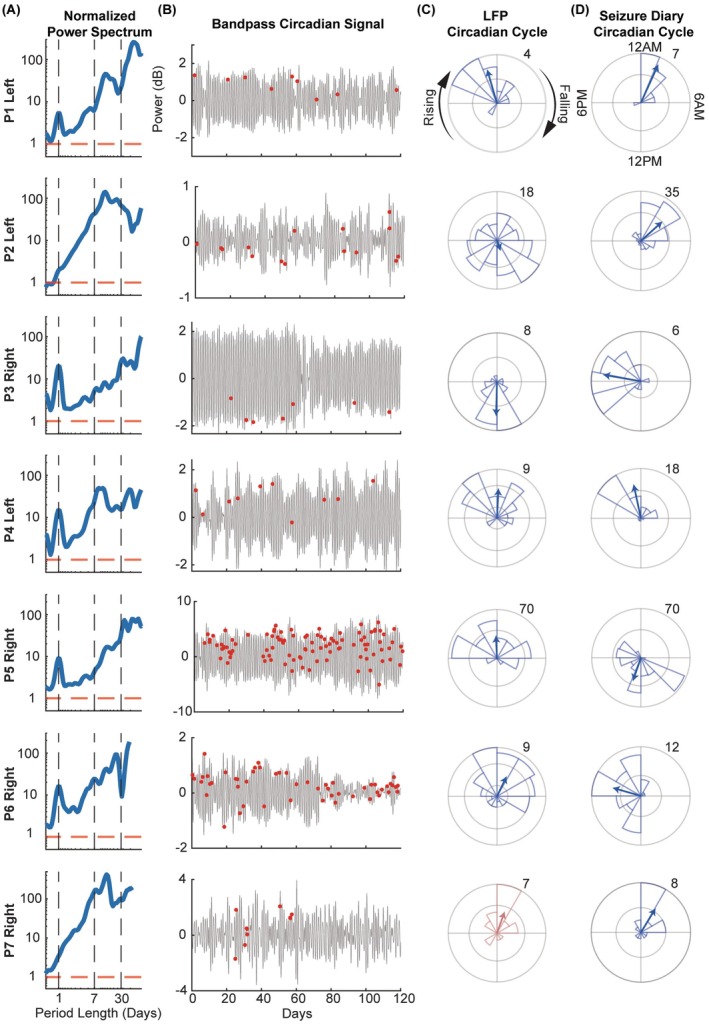
Circadian cycle in ANT and seizure phase‐locking. (A) LFP scalograms averaged over time and normalized to the 95th percentile of normally distributed white‐noise surrogates (red dashed line). Vertical dashed line highlights circadian, weekly, and monthly cycle periods. For each participant example, the hemisphere with the higher *R*‐value between its circadian cycle and seizures is shown. (B) Corresponding bandpass‐filtered circadian tracings with participant‐reported seizure events (red dots) for the first 120 recording days. (C) Polar histogram of seizure occurrence relative to cycle phase. The blue arrow represents the mean resultant angle and R‐value. The number denotes seizure count; for R‐value, the outer ring = 1. P7 (red) did not exhibit a significant circadian cycle in either hemisphere, but seizures were phase‐locked to the circadian period in the right hemisphere (*p* < .05, Rayleigh test). (D) Polar histograms of seizure phase‐locking to a simulated 24‐h sinusoid relative to time of day.

All participants' LFP recordings showed significant circadian and multi‐day cycles (*p* < .05; comparison to white‐noise surrogates, Figure 2A). Self‐reported seizures were significantly phase‐locked to the circadian cycle in at least one hemisphere (*p* < .05, Rayleigh test), indicating seizures clustered at specific phases (Figure 2C). Although P7's power spectrum did not exhibit a local peak at the circadian period, we found significant phase‐locking of seizures to the circadian cycle in the right ANT. Phase‐locking analysis with simulated 24‐h sinusoids further supported a circadian pattern in seizure timing (Figure 2D), suggesting either a diurnal or a nocturnal seizure pattern in most participants.

**FIGURE 3:**
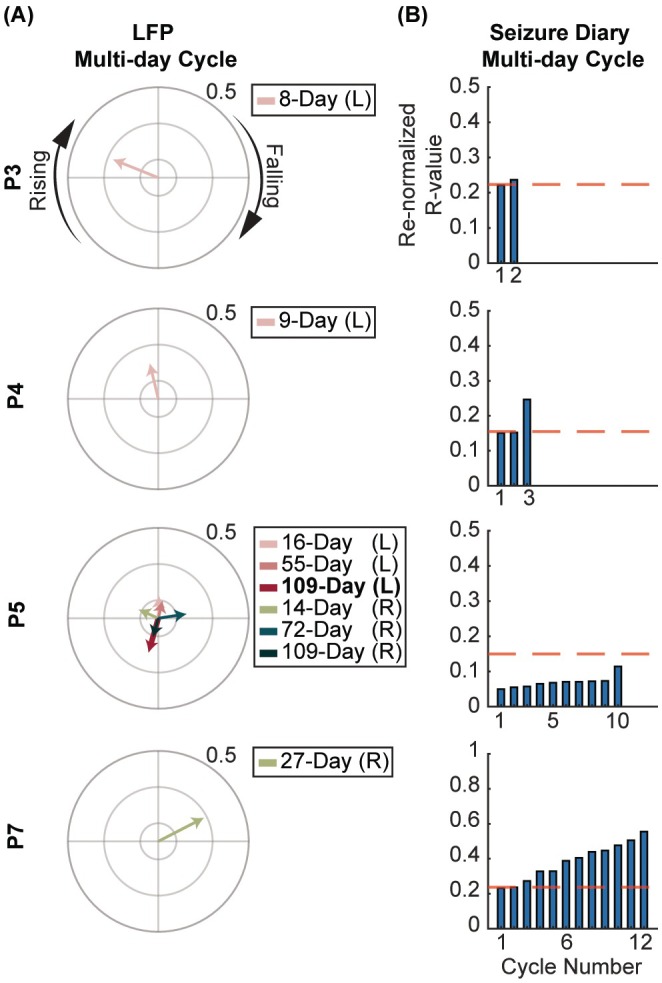
Seizure phase locking to multi‐day cycles in ANT. (A) Polar plots of LFP multi‐day cycles phase‐locked to seizures. The number denotes the R‐value of the outer ring. Recordings from left and right ANTs were analyzed separately and labeled as “L” and “R” in the legends, respectively. For P5, who had multiple cycles detected, the 109‐day (L) cycle had the highest *R*‐value and was thus included in their seizure forecasting model. (B) *R*‐value of multi‐day cycles in each participant's seizure diary. The red dashed line marks the value of the LFP multi‐day cycle with the highest *R*‐value. Since the simulated sinusoids used to identify seizure diary cycles are relative to the arbitrary start of the recording period and not physiologically anchored, comparisons between cycles identified in each approach should focus on the *R*‐value rather than the absolute phase.

Four participants had seizures significantly phase‐locked to certain LFP multi‐day cycles (Figure 3A). Cycles of the same period length were observed across hemispheres in P5, with seizures clustering at similar preferred phases. Multi‐day cycles were also found in the seizure reporting with comparable normalized *R*‐values (Figure 3B), but with distinct cycle periods from LFP cycles (Figure S4).

### 3.3 Seizure forecasting performance

**FIGURE 4:**
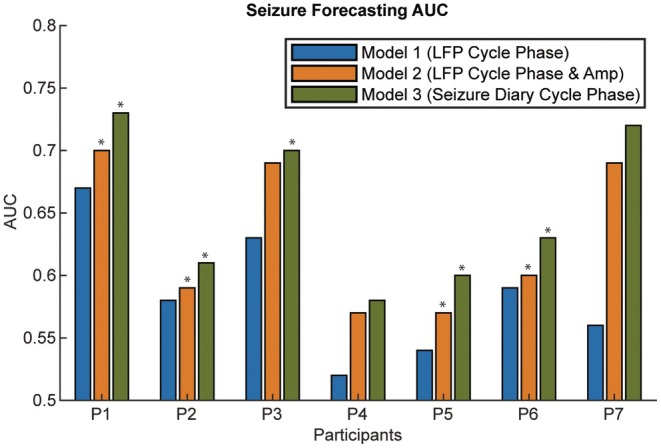
Seizure forecasting AUC. Results of three GPR‐based seizure forecasting methods. Asterisk indicates that AUC is significantly better than surrogates (*p* < .05). Model 1: Using only the phase of LFP cycles. Model 2: Using both phase and amplitude of LFP cycles to forecast seizure risk. Model 3: Using seizure diary–based seizure phase distribution with respect to periodic circadian and multi‐day cycles. P7 did not have any LFP cycles exhibited as a local peak on their LFP power spectrum that were also phase‐locked to seizures. However, the circadian cycle in their right ANT, which was not captured on the power spectrum, was phase‐locked to seizures and used for forecasting here.

Seizure forecasting was performed using three Gaussian process regression (or GPR) models based on: (1) the phase of LFP cycles, (2) both phase and amplitude of LFP cycles, and (3) the phase of seizure cycles derived from seizure diaries. Models based on LFP cycles phase alone did not achieve a performance better than chance in any participant (*p* > .05, threshold determined by the 95th percentile of 200 surrogates). By including the instantaneous amplitude of LFP cycles, four of seven participants' models showed better than chance performance (*p* < .05), indicating that LFP instantaneous cycle amplitude improved performance in all participants. Although forecasting seizures using patterns derived from the seizure diary without neural information achieved the best performance (Figure 4)—five of seven participants performed better than chance (*p* < .05)—the effect size improvement was marginal (Table S3). A Pearson correlation was used to test whether the number of seizures reported correlated with forecasting performance. The result was not statistically significant (*r* = −.55, *p* = .2; Figure S7).

### 3.4 Circadian cycle power and seizure frequency

**FIGURE 5:**
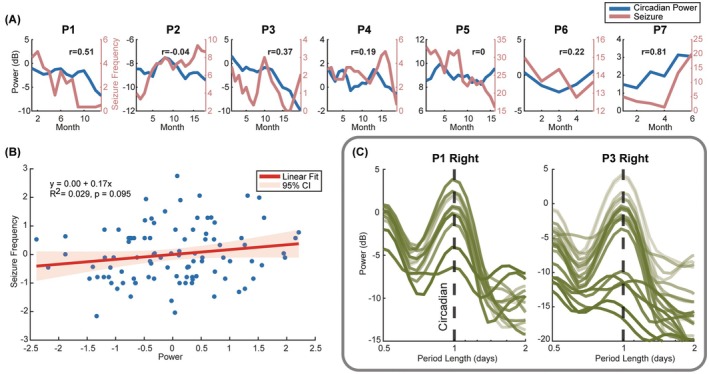
Monthly‐averaged Circadian cycle power and seizure frequency. (A) Three‐month moving average of seizure frequency (red) and LFP circadian cycle power (blue) in the hemisphere with higher power averaged over time. The *x*‐axis shows time since the start of at‐home monitoring of each participant. (B) A linear fit to all participants' monthly seizure frequency and circadian cycle power shows a weak but positive correlation. (C) Monthly power spectrum of P1 and P3 right ANT LFP. Lighter lines indicate that LFP was recorded earlier in the study, and darker lines correspond to later recordings. In both participants, circadian cycle power decreased over time. Despite an impedance normalization having been applied to the analysis, a baseline shift may still be present.

A correlation analysis between the cycle power in the cycle‐dominant hemisphere and seizure frequency revealed a positive correlation in five of seven participants, with several showing moderate correlation coefficients (*R* > .3). A linear regression model fitted to all participants' data showed a positive association between circadian cycle power and seizure frequency (Figure 5B), although this did not reach statistical significance (*p* = .1). In addition, a consistent reduction of the monthly average circadian amplitude over years of recordings was observed in P1 and P3 (Figure 5C).

Recordings from P2 and P5 did not show a correlation between LFP circadian amplitude and seizure frequency (Figure 5A). Of interest, P2 lacked significant non‐uniform circadian modulation in the MI permutation test, whereas P5 exhibited the opposite circadian pattern compared to other participants (Figure S3). Strong cardiac artifacts present in P5's in‐clinic recordings, sampled at 250 Hz, may have contributed to this atypical circadian rhythm.

## 4 DISCUSSION

We found strong circadian and multi‐day cycles in ANT LFP recordings among medication‐resistant epilepsy participants. Self‐reported seizures were found to cluster at specific phases of these cycles. Most participants had seizures clustered at a similar phase of the LFP circadian cycles across hemispheres (Figure S3), suggesting that cycles across sides of ANTs might be modulated by the common underlying physiological or pathological mechanisms. Although the seizure diary showed cyclic patterns similar to LFP power recordings, certain multi‐day cycles in LFP were not reflected by the diary. In all participants, utilizing the instantaneous amplitude of LFP cycles improved forecasting performance. Although the result did not reach statistical significance, a positive correlation was found between the monthly‐averaged power of the LFP circadian cycle and seizure frequency in most participants. This is consistent with a prior RNS (responsive neurostimulation) study that demonstrated the modulation of the circadian cycle in IEA correlated with responder outcome.^30^

Theta/alpha activity in ANT has been reported to be relevant to epilepsy and DBS outcome. Hupalo et al. identified 5–7 Hz theta activity in the ANT of patients with epilepsy,^35^ whereas Scherer et al. showed that ANT stimulation reduces theta‐band power in scalp EEG.^36^ In addition, Chaitanya et al. reported phase‐amplitude coupling between low‐frequency rhythms in the ANT and high‐gamma activity in the SOZ.^37^ Our recent case study also demonstrated seizure reduction associated with suppressed ANT theta/alpha.^34^ Findings here suggest that not only is seizure timing linked to cycles in ANT theta/alpha activity, but also the modulation of the circadian cycle is associated with seizure frequency. Although ANT is involved in the circuit of Papez, a brain network that is commonly involved in epilepsy, short‐term power changes in ANT around seizure events were not consistently found among all participants. This is consistent with findings from previous studies suggesting that not all seizure networks significantly involve ANT,^38^ with different epilepsy types involving different brain regions.^39^

Previous studies have shown that seizures are phase‐locked to cycles in neural and physiological signals, raising the possibility that these rhythms may modulate seizure timing.^20^, ^23^, ^24^ To investigate this further, we examined whether the instantaneous LFP cycle amplitude, a quantitative measurement of the cycle strength, was related to seizure timing. We incorporated the instantaneous cycle amplitude into GPR models and observed enhanced performance across participants, with a few showing a notable increase in AUC, supporting the potential utility of cycle amplitude in forecasting seizure risk. Although the result did not reach statistical significance, a substantial positive correlation between the circadian cycle power and seizure frequency was found in most participants.

The circadian rhythms in epilepsy were often linked to the sleep and awake states.^40^, ^41^, ^42^, ^43^ Studies have found that poor sleep quality is associated with increased seizure likelihood,^4^, ^5^ and ANT‐DBS has been reported to influence sleep,^44^, ^45^, ^46^ which may account for the change in the circadian cycle observed here. However, in some of the participants showing circadian power modulation, we observed that the change is more likely driven by fluctuations in daytime power, rather than a change in the typical rise of theta/alpha power at night (Figure S8). This suggests factors beyond sleep may also influence the circadian modulation observed in the ANT.

### 4.1 Limitations and future directions

This study is solely based on a self‐reported seizure diary, which often struggles with accuracy and consistency.^25^, ^47^ This might explain why no observational change around self‐reported seizure events was found in P2 (Figure S2). Although a recent study found that seizure diaries can be used to estimate the underlying cycles and achieved a similar result to using EEG,^48^ future studies should consider utilizing wearables with the ability to detect seizures to provide better seizure tracking.

Previous studies have shown seizure phase‐locked to the rising phase of IEA multi‐day cycles^20^; the small cohort size limits our ability to assess group‐level phase preferences in thalamic LFP cycles.

Given the distinct cycles captured by the thalamic LFP and seizure diary–based method, we attempted to incorporate cycles identified in both approaches into a GPR‐based forecasting model. However, it did not improve the results consistently across participants (Table S3). One potential cause is that the GPR‐forecasted seizure risk was often dominated by the circadian cycle (Figure S6), and given that LFP was correlated with time of the day in most participants (Table S1), adding a temporally correlated predictor, the LFP circadian cycle, to a model that already included the circadian pattern from the seizure diary is likely not to improve its performance. Furthermore, any systematic biases in seizure‐reporting behavior can introduce periodicity that benefits diary‐based forecasting. In contrast, LFP‐based models are evaluated against these same potentially inaccurate reported seizure times, and any mismatch between actual neural events and biased reporting times will reduce LFP model performance. Future studies with an accurate and unbiased seizure timing should address whether thalamic LFP directly modulates seizure risk or simply reflects a shared underlying structure captured in the seizure diary.

Training a GPR model on a large dataset (multiple cycles) can be computationally expensive, potentially limiting its clinical use. Simpler algorithms that demand fewer computational resources may offer advantages for real‐time applications and clinical translations.^13^, ^49^ Although the GPR model demonstrated strong discriminative discrimination, the resulting probability forecasts were not well calibrated in our implementation (Figure S5), which may limit the direct clinical deployment of this approach.

Beyond these findings, our study was retrospective, and phase‐locking analyses were performed using the entire recording period. Prospective studies aiming to identify long‐term cycles may require extended calibration periods.^14^, ^25^, ^50^, ^51^ Moreover, phase‐locking could change over time due to factors such as medication adjustments.^52^ In addition, the Hilbert transform used for phase and amplitude estimation is non‐causal. Real‐time implementation would require causal phase estimation, potentially via a state‐space model.^53^

Although theta/alpha was chosen based on prior works suggesting it as a biomarker to ANT‐DBS outcomes,^35^, ^36^, ^37^ future works should explore other ANT‐related biomarkers. Our recent work identified that longitudinal suppression in the slow‐gamma frequency is a potential therapeutic biomarker,^29^ and future analyses should examine whether cycles in this frequency band provide complementary forecasting value. In addition, investigating whether the observed cycles reflect fluctuations in aperiodic or periodic components could clarify the underlying mechanisms.^54^ Recent work has also demonstrated that functional connectivity may offer advantages for seizure forecasting,^55^ and future studies should explore whether connectivity dynamics in the ANTs provide predictive information about seizure risk.

## 5 CONCLUSION

Our findings provide evidence that LFP cycles in the ANT are commonly observed in patients with medication‐resistant epilepsy, with self‐reported seizures clustering at specific phases of these cycles. By incorporating the instantaneous amplitude of these cycles into the proposed GPR‐based forecasting models, results showed improved performance against the phase‐only approach, demonstrating the feasibility of seizure forecasting using instantaneous cycle amplitude. Most participants exhibited a substantial association between circadian cycle modulation and clinical outcome, although this effect did not reach statistical significance in this study. Future studies with a larger cohort and a prospective approach may further clarify this association and guide the integration of cycle‐based biomarkers into personalized clinical management of epilepsy.

## AUTHOR CONTRIBUTIONS

Xinbing Zhang and Theoden I. Netoff conceptualized the study. Theoden I. Netoff and Robert A. McGovern served as the principal investigators and supervised the research protocol. Xinbing Zhang, Zachary T. Sanger, and Thomas Lisko coordinated patient recruitment and data collection. Xinbing Zhang, Theoden I. Netoff, and Steffen Ventz contributed to data validation and statistical analysis. Xinbing Zhang drafted the initial manuscript. All authors reviewed and approved the final version of the manuscript.

## CONFLICT OF INTEREST STATEMENT

None of the authors has any conflict of interest to disclose. We confirm that we have read the Journal's position on issues involved in ethical publication and affirm that this report is consistent with those guidelines.

## PATIENT CONSENT

Written informed consent was obtained from all patients for the publication of this study.

## Supporting information


**Figure S1.** Seizure clusters identification.
**Figure S2.** Local field potential (LFP) around self‐reported seizures.
**Figure S3.** Circadian cycles mean resultant vector of both hemispheres.
**Figure S4.** Multi‐day cycles in seizure diary.
**Figure S5.** P1 example of forecasted seizure risk.
**Figure S6.** Autocorrelation and wavelet transform of forecasted probability.
**Figure S7.** Linear regression of seizure count and forecasting performance.
**Figure S8.** Circadian cycle modulation and seizure frequency.
**Figure S9.** Deep‐brain stimulator lead reconstruction.
**Table S1.** Circadian local field potential rhythm mutual information permutation test.
**Table S2.** Seizure phase‐locking to cycles, Rayleigh test, and Omnibus test.
**Table S3.** Seizure forecasting performance.
**Table S4.** Circadian amplitude and seizure frequency correlation *R*‐value.
**Table S5.** 12‐hour cycle amplitude and circadian amplitude/seizure frequency correlation *R‐*value.
**Table S6.** Participant deep‐brain stimulation parameters.

## Data Availability

The data that support the findings of this study are available from the corresponding author upon reasonable request.
