# Supplementary material for: Rhythms in longitudinal thalamic recordings are linked to seizure risk
Source: Epilepsia. 2026 Feb 16;67(5):2559–71. doi: 10.1002/epi.70148 (PMC13179669; doi:10.1002/epi.70148)
Supplement: Supplementary file 1 — Figure S1. Seizure clusters identification. Figure S2. Local field potential (LFP) around self‐reported seizures. Figure S3. Circadian cycles mean resultant vector of both hemispheres. Figure S4. Multi‐day cycles in seizure diary. Figure S5. P1 example of forecasted seizure risk. Figure S6. Autocorrelation and wavelet transform of forecasted probability. Figure S7. Linear regression of seizure count and forecasting performance. Figure S8. Circadian cycle modulation and seizure frequency. Figure S9. Deep‐brain stimulator lead reconstruction. Table S1. Circadian local field potential rhythm mutual information permutation test. Table S2. Seizure phase‐locking to cycles, Rayleigh test, and Omnibus test. Table S3. Seizure forecasting performance. Table S4. Circadian amplitude and seizure frequency correlation R‐value. Table S5. 12‐hour cycle amplitude and circadian amplitude/seizure frequency correlation R‐value. Table S6. Participant deep‐brain stimulation parameters. [file EPI-67-2559-s001.docx]

# *Supplemental Materials*

**
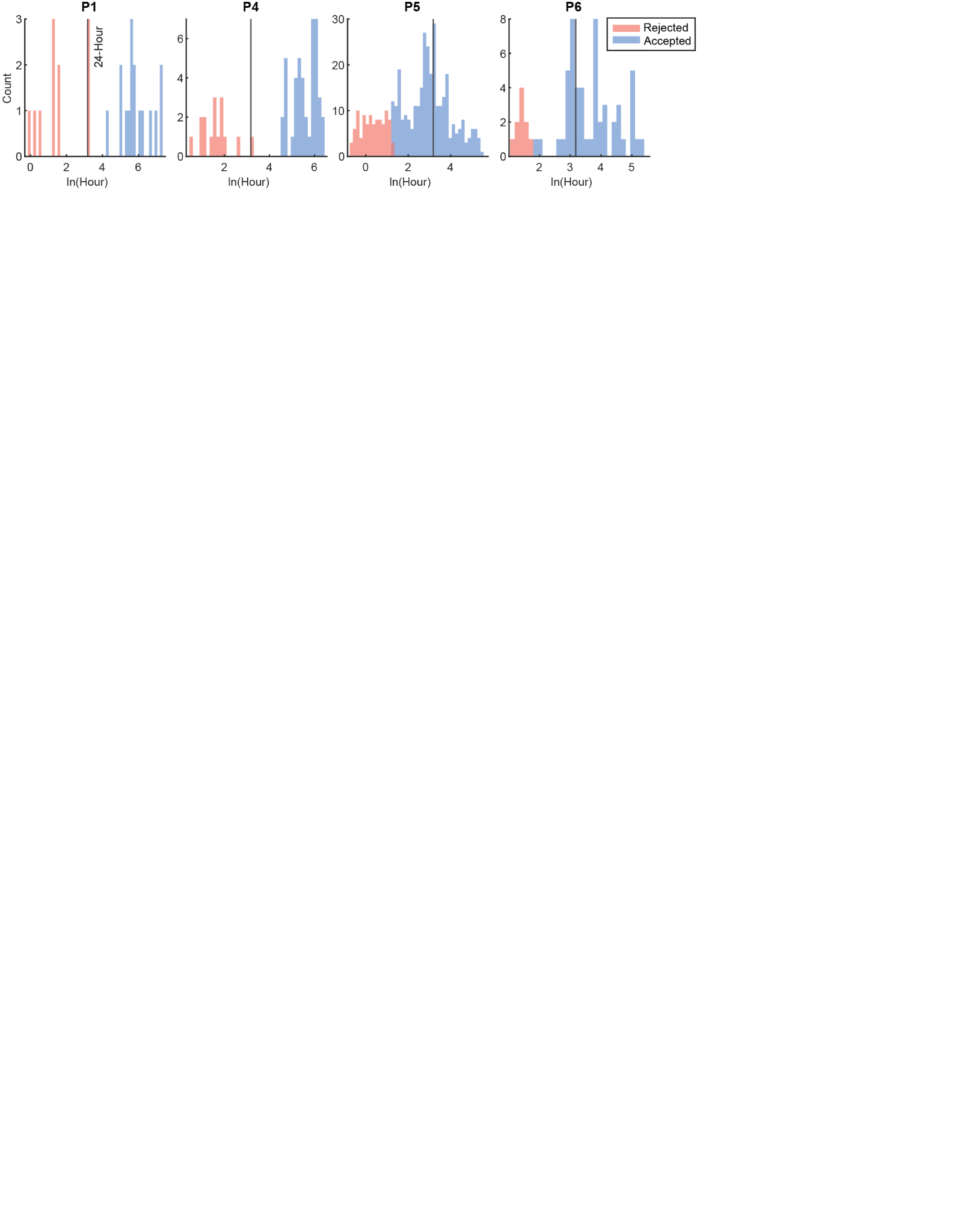
**

**Figure S1 - Seizure Clusters Identification**

Seizures corresponding to the distribution with a shorter inter-seizure interval mean (red) were removed from the forecasting analysis. Possible seizure clusters were found in 4 participants. The x-axis shows the log-transformed inter-seizure interval. All rejected seizure events have inter-seizure intervals shorter than 24 hours.

|  | Left p-value | Right p-value |
| --- | --- | --- |
| **P1** | **<.001** | **<.001** |
| P2 | 0.7 | 0.44 |
| **P3** | **<.001** | **<.001** |
| **P4** | **<.001** | **<.001** |
| **P5** | **<.001** | **<.001** |
| **P6** | **<.001** | **<.001** |
| **P7** | **<.001** | 0.85 |

**Table S1 - Circadian LFP (Local Field Potential) Rhythm Mutual Information Permutation Test**

Mutual information between the hourly mean LFP power recordings and time was compared against 1,000 random permutations of the recordings. All participants except P2 exhibited significance, indicating non-uniform circadian modulation in LFP powers.


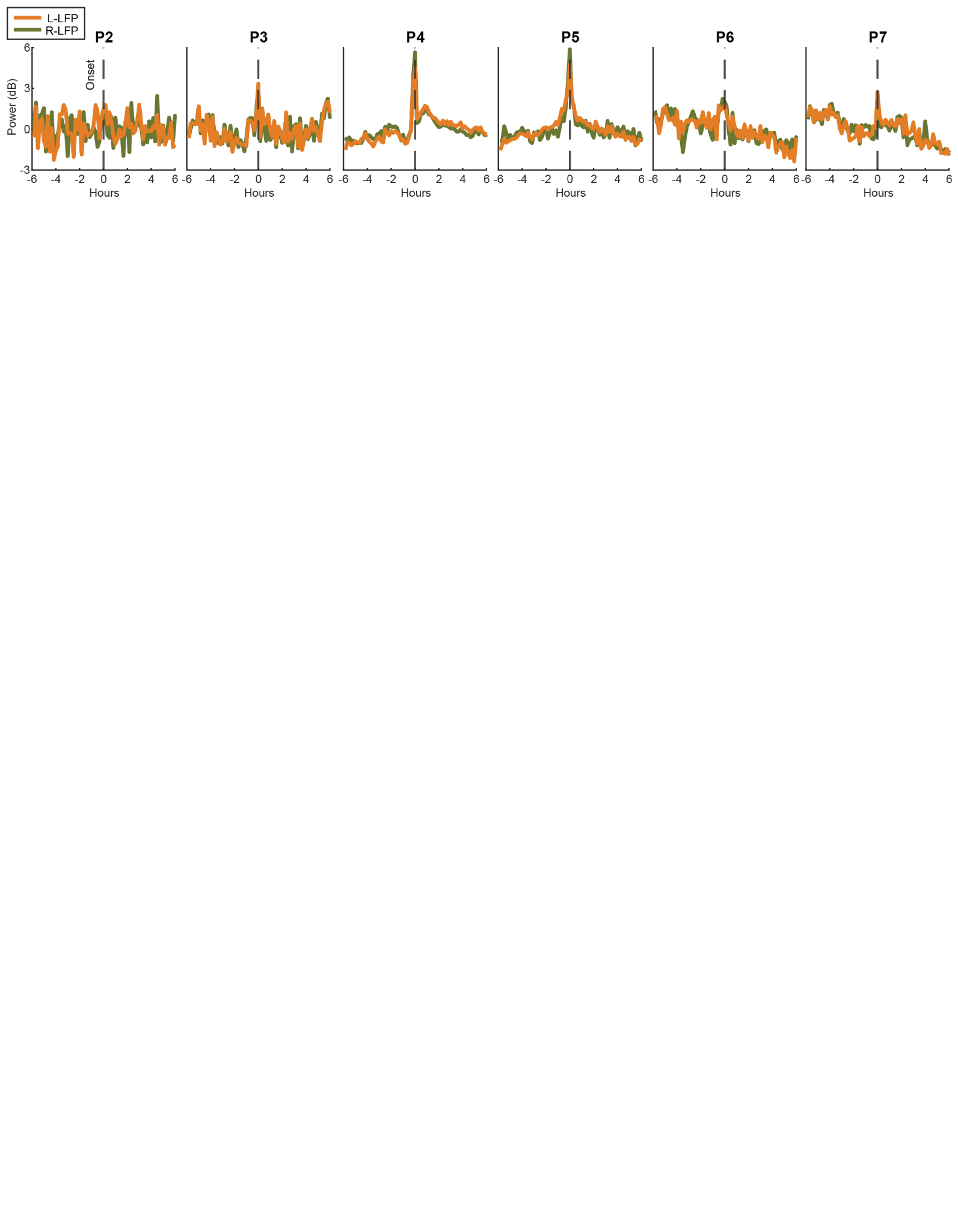


**Figure S2 - LFP Around Self-Reported Seizures**

Increased theta/alpha power was found around self-reported seizures in most participants. However, the power suppression following seizures observed in P1 (Fig. 1) was not seen in other participants’ recordings.

**
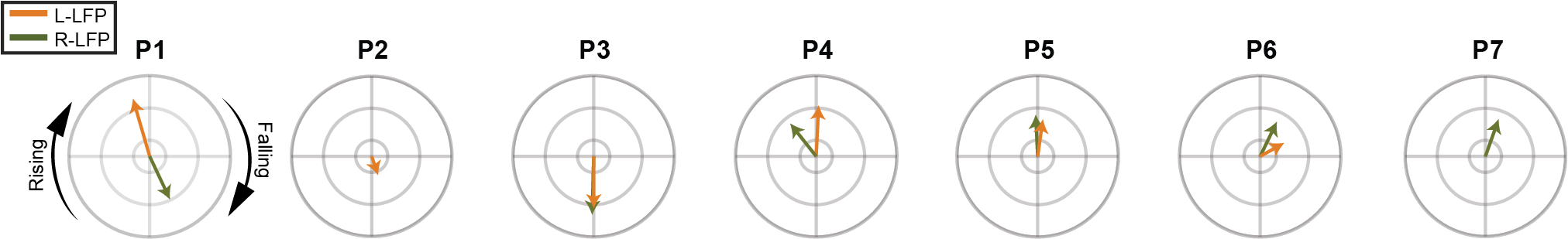
**

**Figure S3 - Circadian Cycles Mean Resultant Vector of Both Hemispheres**

Overall seizure prevalence relative to the LFP (local field potentials) circadian cycle phase in each hemisphere. The green and orange arrows denote the mean resultant angle and phase locking value of left and right ANT (anterior nucleus of thalamus) recordings, respectively. All participants, except P2 and P7, exhibited significant phase-locking in both hemispheres. Most participants had seizures clustered at a similar phase across hemispheres, but P1 exhibited opposite phase preferences between hemispheres.

|  | # of Circadian Cycles | | # of Multiday Cycles | |
| --- | --- | --- | --- | --- |
| Participant | Rayleigh test | Omnibus test | Rayleigh test | Omnibus test |
| P1 | 2 | 2 | 0 | 0 |
| P2 | 1 | 0 | 1 | 1 |
| P3 | 2 | 2 | 1 | 0 |
| P4 | 2 | 2 | 1 | 2 |
| P5 | 2 | 2 | 6 | 4 |
| P6 | 2 | 2 | 0 | 0 |
| P7 | 1 | 0 | 1 | 1 |

**Table S2 - Seizure Phase-Locking to Cycles, Rayleigh test, and Omnibus test**. The number of cycles that were found to be significantly phase-locked with seizures in each participant’s data using the Rayleigh test and the Omnibus test.


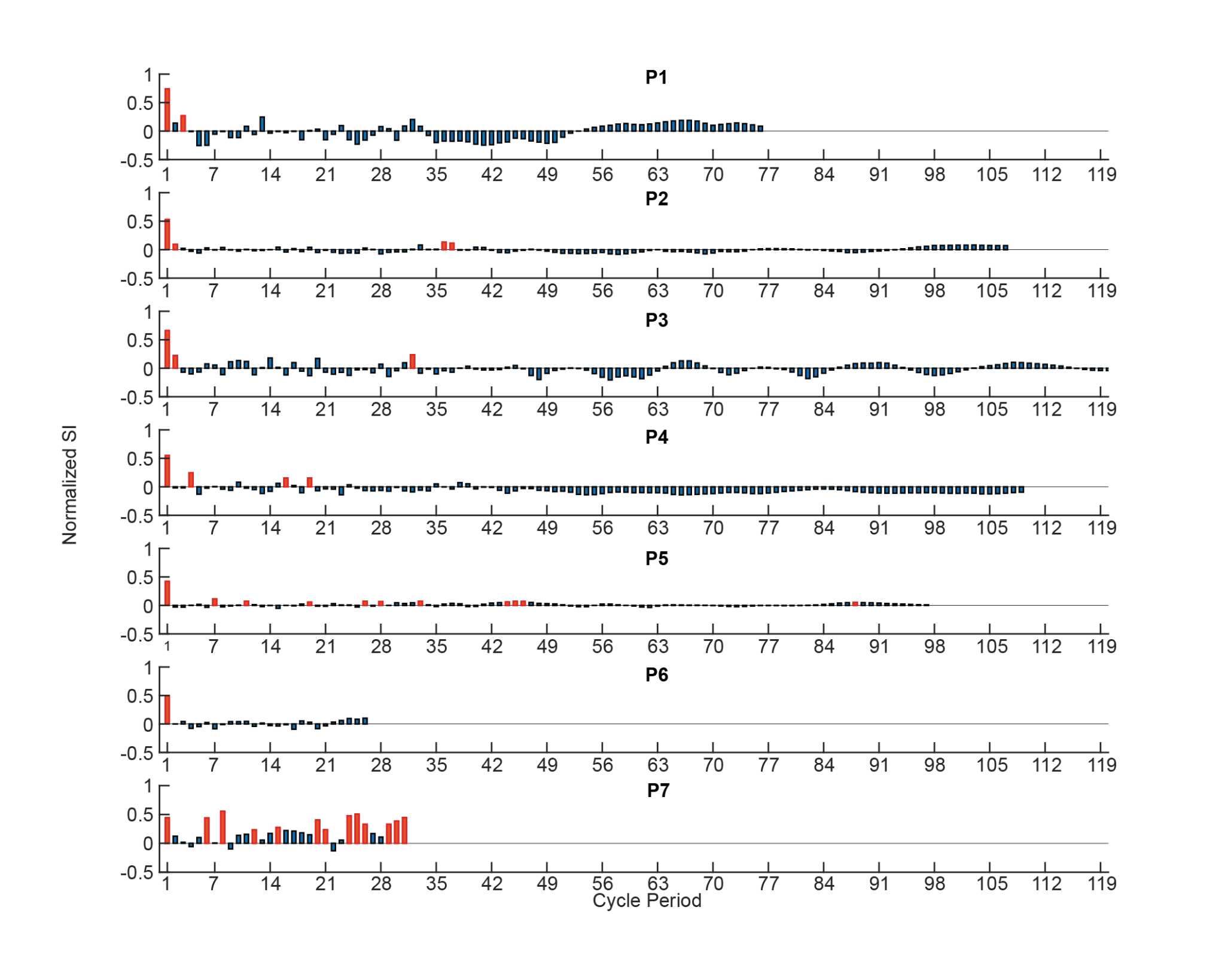


**Figure S4 - Multi-day Cycles in Seizure Diary**

Normalized synchronization index^1^ of self-reported seizures to sinusoids simulated at different cycle periods (Method 2.5). Highlighted bars in red indicate significant phase-locking to seizures using a Rayleigh test.

|  | LFP  Phase Only | | | LFP  Phase + Amp | | | Seizure Patterns | | | LFP Phase + Amp  **AND** Sz Patterns | | | 90-Day Moving Average | | |
| --- | --- | --- | --- | --- | --- | --- | --- | --- | --- | --- | --- | --- | --- | --- | --- |
|  | AUC (p-val; effect size) | AUPRC | BS | AUC (p-val; effect size) | AUPRC | BS | AUC (p-val; effect size) | AUPRC | BS | AUC (p-val; effect size) | AUPRC | BS | AUC | AUPRC | BS |
| P1 (4) | 0.67 (.1; 1.23) | 0.0005 | 0.0007 | 0.7 (.01; 2.04) | 0.001 | 0.0007 | 0.73 (.01; 2.15) | 0.002 | 0.0007 | 0.73 (.01, 2.12) | 0.0025 | 0.0007 | 0.61 | 0.0005 | 0.0003 |
| P2 (7) | 0.58 (.08; 1.46) | 0.0021 | 0.0016 | 0.59 (.02; 2.13) | 0.0021 | 0.0016 | 0.61 (.01; 2.83) | 0.0025 | 0.0016 | 0.62 (.01; 3.71) | 0.0024 | 0.0016 | 0.6 | 0.0022 | 0.0016 |
| P3 (8) | 0.63 (.49; 0.13) | 0.0004 | 0.0004 | 0.69 (.1; 1.23) | 0.0008 | 0.0004 | 0.7 (.04; 1.41) | 0.0008 | 0.0004 | 0.69 (.08; 1.38) | 0.0015 | 0.0004 | 0.72 | 0.0005 | 0.0003 |
| P4 (10) | 0.52 (.75; -0.64) | 0.0006 | 0.0006 | 0.57 (.07; 1.36) | 0.0008 | 0.0006 | 0.58 (.1; 1.28) | 0.0007 | 0.0006 | 0.58 (.11; 1.25) | 0.0007 | 0.0006 | 0.58 | 0.0009 | 0.0006 |
| P5 (11) | 0.54 (.19; 0.94) | 0.0049 | 0.0046 | 0.57 (.01; 3.07) | 0.0069 | 0.0046 | 0.6 (<.01; 4.5) | 0.0068 | 0.0046 | 0.59 (<.01; 3.88) | 0.0065 | 0.0046 | 0.57 | 0.0056 | 0.0046 |
| P6 (14) | 0.59 (.13; 1.12) | 0.0036 | 0.0029 | 0.6 (.03; 1.84) | 0.0044 | 0.0029 | 0.63 (<.01; 2.46) | 0.0048 | 0.0029 | 0.61 (0.02; 1.82) | 0.0039 | 0.0029 | 0.57 | 0.0039 | 0.0029 |
| P7 (1) | 0.56 (.9; -1.34) | 0.0018 | 0.0019 | 0.69 (.21; 0.76) | 0.0024 | 0.0019 | 0.72 (0.13; 1.15) | 0.0108 | 0.0019 | 0.74 (0.06; 1.46) | 0.0196 | 0.0019 | 0.74 | 0.0036 | 0.0009 |

**Table S3 - Seizure Forecasting Performance**

Numbers in the parenthesis shows the p-value and effect size of models trained using actual data against 200 surrogate models with randomly shuffled seizure-to-seizure intervals. The 95th percentile of 200 surrogates’ AUC score was used as the better-than-chance threshold. In addition to the Gaussian process regression-based models proposed in the study, we also included the forecasting performance of a 90-day moving average model^2,3^. AUC - Area under the curve of sensitivity vs. corrected proportion of time-in-warning. AUPRC - Area under the precision-recall curve. BS - Brier score.

**
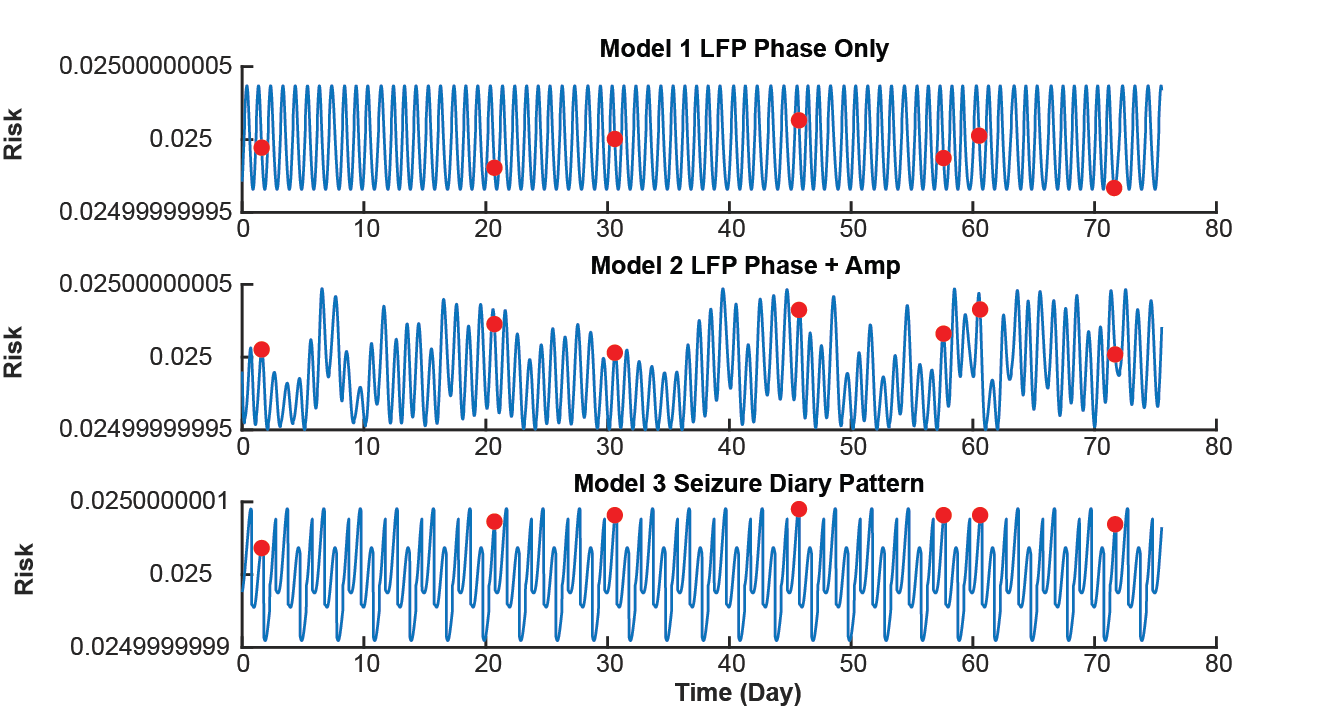
**

**Figure S5 - P1 Example of Forecasted Seizure Risk**

Each panel represents the seizure risk forecasted by each model. Note that only the circadian cycle of thalamic LFP was found to be significantly phase-locked with seizures, while a 3-day seizure diary pattern was found in P1 and incorporated into Model 3 here. Although a Gaussian process regression model captures the relationship between phase, amplitude, and seizure risk, the forecasted probability centers around the mean seizure risk, potentially indicating a lack of calibration for actual clinical use.

**
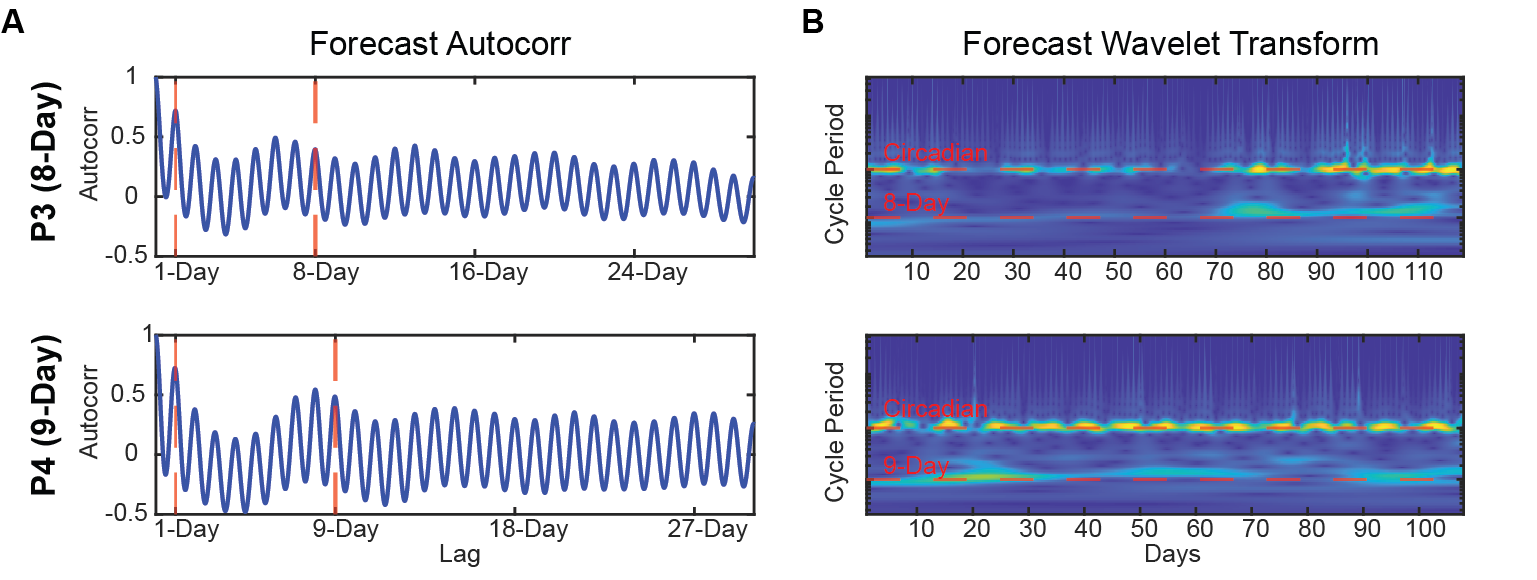
**

**Figure S6 - Autocorrelation and Wavelet Transform of Forecasted Probability**

Two participant examples with multiday cycles incorporated into the seizure forecasting model show forecast was mainly driven by their circadian cycle. An 8-day cycle for P3 and a 9-day cycle for P4. (A) Autocorrelation of a 30-day forecasted seizure risk. Vertical dashed lines indicate their circadian and multiday cycle periods. (B) Continuous Wavelet transform generated spectrograms of forecasted seizure risk. Horizontal dashed lines highlight their circadian and multiday cycle periods. A brighter color indicates a stronger amplitude of the corresponding cycle.


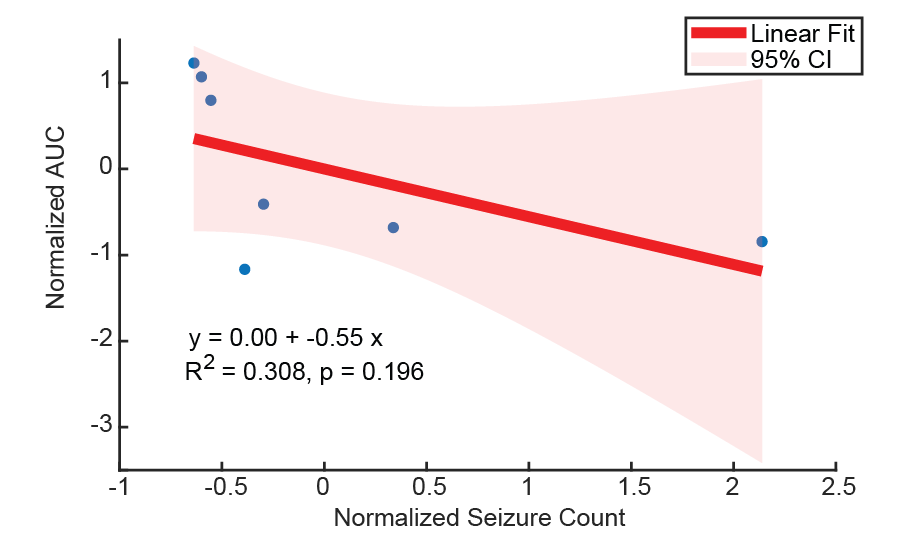


**Figure S7 - Linear Regression of Seizure Count and Forecasting Performance**

A linear regression model was fitted to the number of seizure events reported by each participant and their corresponding forecasting performance using Model 2 (LFP cycle phase + amplitude). No significant correlation was found (p=0.2).


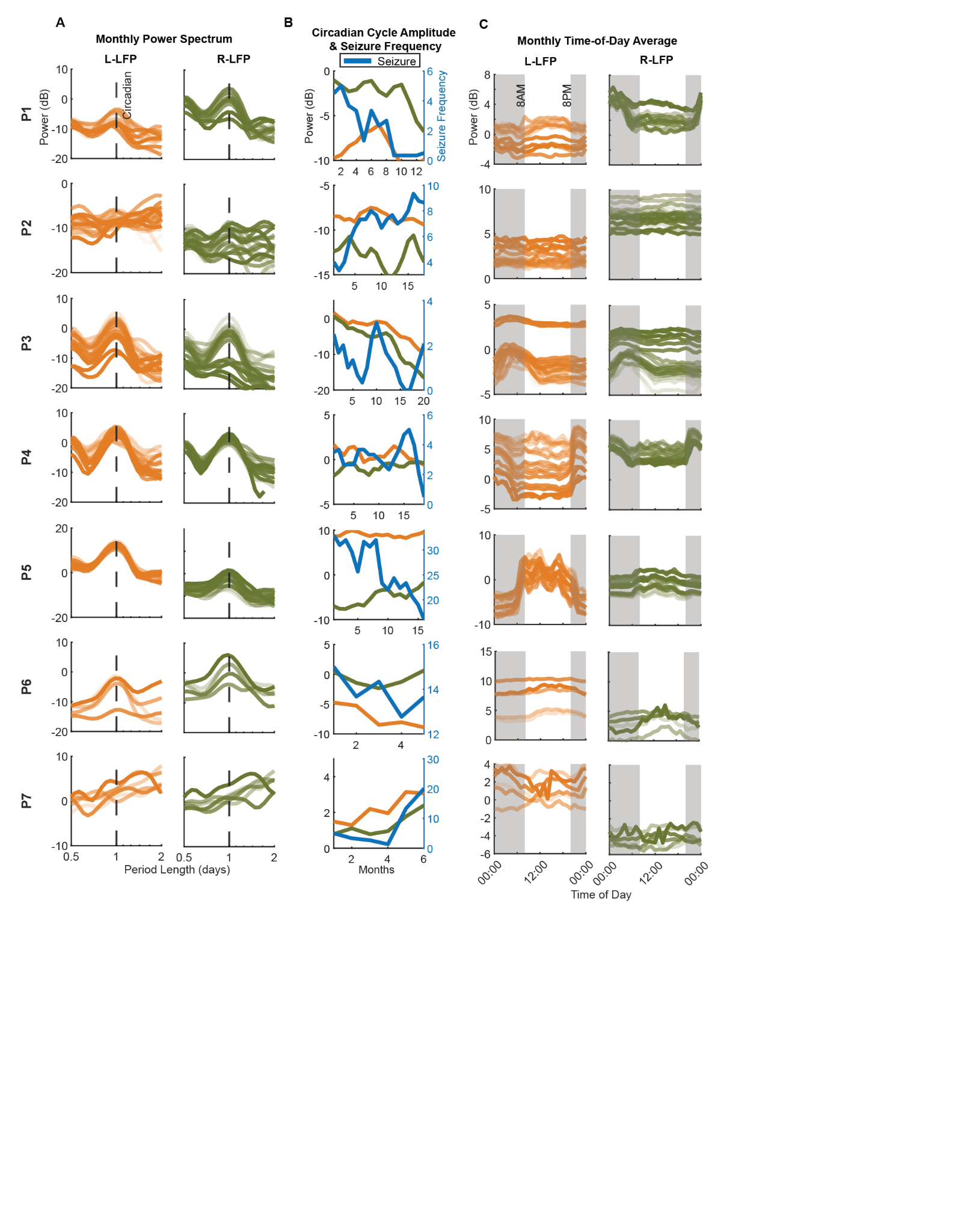


**Figure S8 - Circadian Cycle Modulation and Seizure Frequency**

(A) Monthly scalograms of left and right ANT LFP powers averaged over time. Results were normalized by the impedance of recording contacts. Lines with a more transparent color indicate LFP collected earlier in the study, and vice versa. (B) 3-month moving average of circadian cycle amplitude modulation in left (orange) and right (green) hemispheres, along with seizure frequency (blue). The x-axis shows time since the start of at-home monitoring of each participant. (C) LFP averaged by time of day in each month. The same color code in (A) applies here. P1, P3, and P4 demonstrated the circadian pattern with increased activity during night (shaded in grey). Despite an impedance normalization having been applied, a potential baseline shift still exists in some participants’ results.

| Participant  (Dominant Side) | Left ANT | Right ANT |
| --- | --- | --- |
| P1 (R) | 0.34 | 0.51 |
| P2 (L) | -0.04 | -0.15 |
| P3 (L) | 0.37 | 0.37 |
| P4 (L) | 0.19 | -0.29 |
| P5 (L) | 0 | -0.82 |
| P6 (R) | 0.48 | 0.22 |
| P7 (L) | 0.81 | 0.96 |

**Table S4 - Circadian Amplitude and Seizure Frequency Correlation R-value**

Each row contains the 3-point moving average correlation results of LFP in each hemisphere with seizure frequency. The hemisphere with the higher circadian power is indicated in parentheses next to the participant number.

|  | w/ Seizure Frequency | | w/ Circadian Amplitude | |
| --- | --- | --- | --- | --- |
|  | Left ANT | Right ANT | Left ANT | Right ANT |
| P1 (R) | -0.09 | 0.4 | 0.84 | 0.94 |
| P2 (L) | 0.56 | -0.25 | 0.33 | 0.77 |
| P3 (L) | 0.2 | 0.32 | 0.97 | 0.98 |
| P4 (L) | 0.17 | **0.41** | 0.48 | **-0.2** |
| P5 (L) | 0.54 | -0.05 | 0.45 | 0.47 |
| P6 (L) | 0.6 | 0.63 | 0.98 | 0.82 |
| P7 (R) | 0.46 | 0.91 | 0.83 | 0.91 |

**Table S5 - 12-hour Cycle Amplitude and Circadian Amplitude/Seizure Frequency Correlation R-value**

In addition to the circadian cycle, we observed a strong 12-hour cycle in several participants’ recordings. Most participants showed strong correlations between their circadian and 12-hour cycles in ANTs. However, a negative correlation was found in P4’s right hemisphere (red & bold). This indicates that the circadian and 12-hour cycles could be modulated by different underlying mechanisms. A positive correlation between 12-hour cycle power and seizure frequency was found in most participants, consistent with our findings on the circadian cycle power modulation.

**
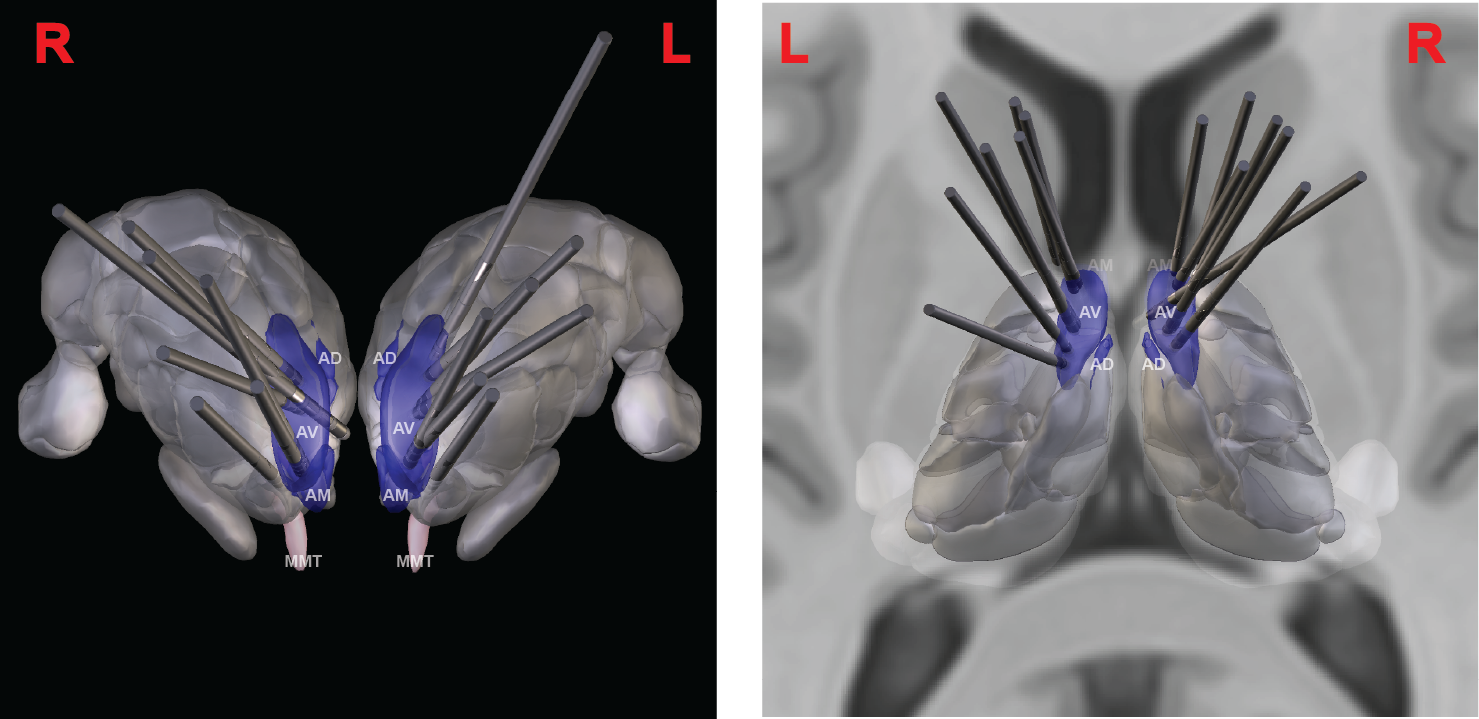
**

**Figure S9 - DBS Lead Reconstruction**

Coronal and axial views of all participants’ bilateral lead placements reconstructed using LeadDBS^4^. The Morel atlas is used for visualization^5^. “L” and “R” indicate hemisphere side. Blue-shaded tissue represents the ANT: AD - anteriordorsal, AV - anteriorventral, AM - anteriormedial. The mammillothalamic tract (MMT) is highlighted in pink.

| Participant | Time  (Month) | CircadianAmp  Dominant | Seizure  Frequency | Amplitude (mA) | Pulse Width (us) | Frequency (Hz) |
| --- | --- | --- | --- | --- | --- | --- |
| **1** | 1 | 0.55 | 5.00 | 1.5 | 120 | 145 |
|  | 2 | 0.25 | 6.33 | 1.5 | 120 | 145 |
|  | 3 | 0.34 | 5.00 | 3 | 90 | 145 |
|  | 4 | 0.83 | 4.33 | 3 | 90 | 145 |
|  | 5 | 1.03 | 1.33 | 3 | 90 | 145 |
|  | 6 | 0.78 | 3.33 | 3 | 90 | 145 |
|  | 7 | -0.14 | 2.33 | 3 | 90 | 145 |
|  | 8 | -0.49 | 3.00 | 4.3 | 50 | 125 |
|  | 9 | -0.25 | 1.00 | 4.3 | 50 | 125 |
|  | 10 | -0.09 | 1.00 | 4.3 | 50 | 125 |
|  | 11 | -0.40 | 1.00 | 3 | 90 | 145 |
|  | 12 | -1.09 | 0.67 | 4.3 | 50 | 125 |
|  | 13 | -1.44 | 1.00 | 4.3 | 50 | 125 |
| **2** | 1 | 0.37 | 4.00 | 4 | 90 | 145 |
|  | 2 | 0.05 | 3.33 | 4 | 90 | 145 |
|  | 3 | 0.02 | 4.00 | 5 | 60 | 125 |
|  | 4 | 0.03 | 5.67 | 5 | 60 | 125 |
|  | 5 | 0.33 | 6.67 | 4 | 90 | 145 |
|  | 6 | 1.07 | 7.34 | 4 | 90 | 145 |
|  | 7 | 1.62 | 7.34 | 4 | 90 | 145 |
|  | 8 | 1.57 | 8.00 | 4 | 90 | 145 |
|  | 9 | 0.60 | 7.67 | 4 | 90 | 145 |
|  | 10 | -0.48 | 6.67 | 4 | 90 | 145 |
|  | 11 | -0.78 | 7.34 | 4 | 90 | 145 |
|  | 12 | -0.81 | 7.67 | 4 | 90 | 145 |
|  | 13 | -0.64 | 7.00 | 4 | 90 | 145 |
|  | 14 | -0.48 | 7.34 | 4 | 90 | 145 |
|  | 15 | -0.23 | 8.00 | 4 | 90 | 145 |
|  | 16 | -0.38 | 9.34 | 4 | 90 | 145 |
|  | 17 | -0.69 | 8.76 | 4 | 90 | 145 |
|  | 18 | -0.96 | 8.64 | 4 | 90 | 145 |
| **3** | 1 | 1.92 | 2.50 | 2 | 90 | 145 |
|  | 2 | 1.39 | 1.67 | 3 | 90 | 145 |
|  | 3 | 1.28 | 2.00 | 3 | 90 | 145 |
|  | 4 | 0.67 | 1.00 | 1.7 | 140 | 165 |
|  | 5 | 0.97 | 1.33 | 1.7 | 140 | 165 |
|  | 6 | 0.19 | 0.67 | 1.7 | 140 | 165 |
|  | 7 | 0.01 | 0.33 | 1.7 | 140 | 165 |
|  | 8 | -0.27 | 1.00 | 1.7 | 140 | 165 |
|  | 9 | 0.00 | 2.33 | 1.7 | 140 | 165 |
|  | 10 | 0.14 | 3.00 | 1.7 | 140 | 165 |
|  | 11 | 0.00 | 2.33 | 2.9 | 60 | 165 |
|  | 12 | -0.02 | 1.67 | 2.9 | 60 | 165 |
|  | 13 | -0.35 | 1.33 | 2.9 | 60 | 165 |
|  | 14 | -0.68 | 1.00 | 2.9 | 60 | 165 |
|  | 15 | -0.98 | 0.33 | 2.9 | 60 | 165 |
|  | 16 | -1.07 | 0.00 | 2.9 | 60 | 165 |
|  | 17 | -1.18 | 0.00 | 2.9 | 60 | 165 |
|  | 18 | -1.11 | 0.67 | 2.9 | 60 | 165 |
|  | 19 | -0.67 | 1.38 | 2.9 | 60 | 165 |
|  | 20 | -0.35 | 2.07 | 2.9 | 60 | 165 |
| **4** | 1 | 0.61 | 3.50 | 2.7 | 90 | 125 |
|  | 2 | 0.26 | 3.67 | 2.7 | 90 | 125 |
|  | 3 | 0.09 | 2.67 | 2.7 | 90 | 125 |
|  | 4 | 1.33 | 2.67 | 2.7 | 90 | 125 |
|  | 5 | 1.45 | 2.67 | 2.7 | 90 | 125 |
|  | 6 | 1.16 | 3.67 | 2.5 | 90 | 145 |
|  | 7 | 0.02 | 3.67 | 2.5 | 90 | 145 |
|  | 8 | -0.09 | 3.33 | 2.7 | 90 | 125 |
|  | 9 | -0.28 | 3.33 | 2.7 | 90 | 125 |
|  | 10 | -0.45 | 3.00 | 2.7 | 90 | 125 |
|  | 11 | -0.46 | 2.67 | 2.7 | 90 | 125 |
|  | 12 | -0.47 | 2.33 | 2.7 | 90 | 125 |
|  | 13 | -0.47 | 3.00 | 3.3 | 60 | 125 |
|  | 14 | -0.48 | 3.67 | 3.3 | 60 | 125 |
|  | 15 | -0.49 | 4.67 | 2.7 | 90 | 125 |
|  | 16 | -0.50 | 5.00 | 2.7 | 90 | 125 |
|  | 17 | -0.51 | 4.00 | 2.7 | 90 | 125 |
|  | 18 | -0.51 | 2.00 | 2.7 | 90 | 125 |
|  | 19 | -0.51 | 0.50 | 2.7 | 90 | 125 |
| **5** | 1 | -0.53 | 66.52 | 4.5 | 90 | 145 |
|  | 2 | -0.51 | 62.35 | 5.9 | 120 | 125 |
|  | 3 | -0.22 | 60.35 | 4.5 | 90 | 145 |
|  | 4 | 0.89 | 44.34 | 5.9 | 120 | 125 |
|  | 5 | 1.47 | 36.34 | 5.9 | 120 | 125 |
|  | 6 | 1.64 | 50.01 | 4.2 | 120 | 125 |
|  | 7 | 0.42 | 46.01 | 5.9 | 60 | 125 |
|  | 8 | -0.24 | 45.34 | 5.9 | 60 | 125 |
|  | 9 | -0.80 | 32.34 | 5.9 | 60 | 125 |
|  | 10 | -0.80 | 32.01 | 5.9 | 60 | 125 |
|  | 11 | -0.70 | 31.01 | 4.2 | 120 | 125 |
|  | 12 | -0.14 | 25.67 | 4.2 | 120 | 125 |
|  | 13 | 0.11 | 23.01 | 4.2 | 120 | 125 |
|  | 14 | 0.10 | 20.67 | 4.8 | 90 | 125 |
|  | 15 | -0.29 | 19.00 | 4.8 | 90 | 125 |
|  | 16 | -0.41 | 16.00 | 4.2 | 120 | 125 |
| **6** | 1 | -0.65 | 15.00 | 4 | 90 | 145 |
|  | 2 | -0.39 | 13.67 | 4 | 90 | 145 |
|  | 3 | -0.34 | 14.34 | 4 | 90 | 145 |
|  | 4 | 0.43 | 12.77 | 4 | 90 | 145 |
|  | 5 | 0.58 | 13.65 | 4 | 90 | 145 |
| **7** | 1 | -0.27 | 5.00 | 1 | 90 | 145 |
|  | 2 | -0.52 | 3.33 | 1 | 90 | 145 |
|  | 3 | -0.42 | 2.67 | 1 | 90 | 145 |
|  | 4 | -0.46 | 1.33 | 1 | 90 | 145 |
|  | 5 | 0.52 | 13.35 | 1 | 90 | 145 |
|  | 6 | 0.87 | 20.02 | 1 | 90 | 145 |

**Table S6 - Participant Deep Brain Stimulation Parameters**

The dominant hemisphere was selected based on which hemisphere exhibited a stronger circadian cycle across time. Circadian amplitude was z-scored.

**References**

1. Andrzejak RG, Espinoso A, García-Portugués E, Pewsey A, Epifanio J, Leguia MG, et al. High expectations on phase locking: Better quantifying the concentration of circular data. Chaos Interdiscip J Nonlinear Sci. 2023 Sep 27;33(9):091106.

2. Chang CY, Moss R, Westover MB, Goldenholz DM. Rigorous evaluation of five models for e-diary-only seizure forecasting: Retrospective and prospective datasets do not outperform the Napkin method. Epilepsia. 2025 Oct 14;

3. Stirling RE, Brinkmann BH, Freestone DR, Karoly PJ. Tracking seizure cycles beats a prospective moving average: Commentary on “Rigorous evaluation of five e-diary alone seizure forecasting tools.” Epilepsia. 2025 Dec 31;

4. Neudorfer C, Butenko K, Oxenford S, Rajamani N, Achtzehn J, Goede L, et al. Lead-DBS v3.0: Mapping Deep Brain Stimulation Effects to Local Anatomy and Global Networks. NeuroImage. 2023 Mar;268:119862.

5. Krauth A, Blanc R, Poveda A, Jeanmonod D, Morel A, Székely G. A mean three-dimensional atlas of the human thalamus: Generation from multiple histological data. NeuroImage. 2010 Feb 1;49(3):2053–62.
